# Failed preoperative vacuum bell therapy does not affect outcomes following minimally invasive repair of pectus excavatum

**DOI:** 10.1007/s00383-021-04963-6

**Published:** 2021-07-16

**Authors:** J. L. Muff, L. C. Guglielmetti, S. J. Gros, L. Buchmüller, G. Frongia, F. -M. Haecker, S. G. Holland-Cunz, T. de Trey, Raphael N. Vuille-dit-Bille

**Affiliations:** 1 Department of Pediatric Surgery, University Children’s Hospital of Basel, Spitalstrasse 33, 4056 Basel, Switzerland; 2 Department of Visceral and Thoracic Surgery, Cantonal Hospital of Winterthur, Winterthur, Switzerland; 3 Division of Pediatric Surgery, Department of General, Visceral and Transplantation Surgery, University Hospital Heidelberg, Heidelberg, Germany; 4 Department of Pediatric Surgery, Children′s Hospital of Eastern Switzerland, St.Gallen, Claudiusstrasse 6, CH-9006, St.Gallen, Switzerland; 5 Faculty of Medicine, University of Basel, 4056 Basel, Switzerland

**Keywords:** Pectus excavatum, Minimal invasive repair of pectus excavatum, MIRPE, Nuss procedure, Vacuum bell therapy

## Abstract

**Purpose:**

It is unknown if failed preoperative vacuum bell (VB) treatment in patients undergoing minimally invasive repair of pectus excavatum (MIRPE), delays repair and/or affects postoperative outcomes.

**Methods:**

A retrospective data analysis including all consecutive patients treated at one single institution undergoing MIRPE was performed between 2000 and 2016. Patients were stratified into preoperative VB therapy versus no previous VB therapy.

**Results:**

In total, 127 patients were included. Twenty-seven (21.3%) patients had preoperative VB treatment for 17 months (median, IQR 8–34). All 27 patients stopped VB treatment due to the lack of treatment effect. Eight (47.1%) of 17 assessed VB patients showed signs of skin irritation or hematoma. VB treatment had no effect on length of hospital stay (*p* = 0.385), postoperative complications (*p* = 1.0), bar dislocations (*p* = 1.0), and duration of bar treatment (*p* = 0.174). Time spent in intensive care unit was shorter in patients with VB therapy (*p* = 0.007). Long-term perception of treatment including rating of primary operation (*p* = 0.113), pain during primary operation (*p* = 0.838), own perspective of look of chest (*p* = 0.545), satisfaction with the procedure (*p* = 0.409), and intention of doing surgery again (*p* = 1.0) were not different between groups.

**Conclusions:**

Failed preoperative VB therapy had no or minimal effect on short-term outcomes and long-term perceptions following MIRPE.

## Introduction

Pectus excavatum (PE) or funnel chest is the most common congenital anterior thoracic wall deformity accounting for > 90% of congenital chest wall deformities [1]. It is estimated to occur in 1 in 400 births [2], occurring three to five times more often in males [3]. Clinical manifestations may comprise dyspnea, fatigue during activities, decreased endurance, anterior chest pain and tachycardia. Furthermore, the disfigurement can lead to social withdrawal, low self-esteem, harassment by peers, anxiety, and feelings of stigma and shame, resulting in reduced mental quality of life [4–8].

While different treatment techniques have been described in the past, minimally invasive repair of pectus excavatum (MIRPE) developed by Dr. D. Nuss in 1987 and subsequently presented at the American Pediatric Surgery Association Congress in 1997, presently reflects the standard therapy for PE [9, 10]. In mild to moderate cases and/or in patients reluctant to operative therapy, vacuum bell (VB) therapy reflects an alternative treatment option to surgery [8, 11–14]. VB therapy consists of a suction cup, attached to the anterior chest, covering the depression of the sternum. Applying negative pressure up to 15% below atmospheric pressure, the sternum and ribs can be lifted within 1–2 min [11]. Suggested application time of vacuum bell ranges from 30 min twice per day up to several hours daily with a recommended 12–15-month treatment course. Contraindications to VB therapy consist of skeletal disorders such as osteogenesis imperfecta and Glisson’s disease, vasculopathies, coagulopathies and some cardiac disorders [12]. Side effects of VB therapy comprise subcutaneous hematoma, petechial bleeding, dorsalgia, and momentary paresthesia of the arms during application [14]. Long-term follow-up on VB patients has yet to be performed.

VB therapy has been propagated as a potentially useful “pre-treatment” to surgery [14] or “adjunct treatment” to MIRPE [11]. However, previous studies have failed to include a comparison of patients with preoperative VB therapy to patients with direct surgery. It is unknown if failed preoperative VB treatment in patients undergoing minimally invasive repair of PE delays repair and/or affects postoperative outcomes. Our hypothesis is that VB therapy has no or minimal effect on these outcomes and can be offered safely prior to surgery. The present study hence assesses intra- and postoperative outcomes following MIRPE in patients with discontinued preoperative VB treatment compared to direct MIRPE.

## Materials and methods

This is a retrospective cohort study assessing the outcomes of VB therapy preceding MIRPE compared to MIRPE alone. Patients included in this study were consecutively operated at one single institution (The University Children’s Hospital Basel) between February 2000 and December 2016. The operations were performed by the same group of board-certified pediatric surgeons. Patients treated with vacuum bell only were not included in the present study.

### Minimally invasive repair of pectus excavatum

MIRPE was performed in supine position with 90° abducted arms using bilateral vertical incisions under right-sided thoracoscopic guidance. Elevation of the sternum was performed if it was the surgeon’s preference. An Endo-Kittner (Auto-Suture, US Surgical Corp, Norwalk, CT) was used to perform blunt dissection across the mediastinum. The Nuss bar introducer was then inserted from left to right, pulling a tracheostomy tape across the mediastinum. The pectus bar was tied to the tape and pulled across the mediastinum under direct vision. The bar was rotated into position and anchored using zero, one or two stabilizer and/or sutures. This was followed by a final inspection of the mediastinum, reduction of the pneumothorax using a water-seal drain, sutures and post-procedure chest radiography.

### Follow-up

Routine clinical follow-ups were conducted in all patients within 28 days after surgery. In addition, long-term follow-up by phone interview was performed 9 years (median) after the primary operation (ranging from 4 to 21 years).

### Outcomes

Assessed outcomes consisted of age, gender, Haller index, funnel depth, operation time, duration of postoperative stay, time spent in the intensive care unit (ICU), postoperative complications, side of stabilizers, number of bars, intraoperative chest elevation, time to bar removal, bar dislocations and long-term satisfaction with the result. The aforementioned data were retrospectively assessed from hospital internal servers and interviews were conducted over phone.

### Statistics

Study data were collected and managed using REDCap electronic data capture tools hosted at University Children’s Hospital Basel [15, 16]. Descriptive statistics were used for the overview of patients’ characteristics. Median and interquartile range (IQR) were reported for non-normally distributed data. Mann–Whitney *U* test was used for collating the two groups. Categorical variables were reported as frequencies (%) and compared using Pearson’s χ^2^ test or Fisher’s exact test. *p *values < 0.05 (two-tailed) were considered statistically significant. Normality was assessed using the Shapiro–Wilk test. IBM SPSS Statistics (Version: 25, IBM Corp., Armonk, N.Y.) was used for performing all statistical analyses.

## Ethics

The study was approved by the local ethics committee (reference number: 2016-02211) and informed consent was obtained by patients’ caregivers.

## Results

### Patients’ characteristics

In total 127 patients were included. Twenty-seven (21.3%) patients had preoperative VB treatment for 17 months with a minimum of 3 months and a maximum of 78 months (median, IQR 8–34). Table 1 illustrates patients’ characteristics. As expected, there was a dominance of male patients in both groups (74.1% VB, 83% non-VB; *p* = 0.405). Median age at operation was 15.8 (IQR 14.6–17.8) years in VB and 15.9 (IQR 14.4–16.8) years in non-VB patients (*p* = 0.393). Characteristic of PE was similar in respect to Haller index (i.e. the transverse diameter of the chest divided by the anterior–posterior distance gathered from an axial CT scan) [17] (3.7 VB, IQR 3.2–4.4 vs. 3.5 non-VB, IQR 2.9–4.3; *p* = 0.256), funnel depth (3 cm VB, IQR 2.5–3.7 vs. 3.1 non-VB, IQR 2.6–4; *p* = 0.721) and presence of symmetry (66.7% VB vs. 67% non-VB; *p* = 1.0). Fifty patients (39.4%) had a secondary diagnosis (14 (51.9%) VB vs. 36 (36%) non-VB; *p* = 0.183), mostly consisting of either kyphosis, scoliosis, Marfan syndrome or a combination of these three.

**Table 1:** Characteristics of patients and pectus excavatum

Variable	VB (*n* = 27)	non-VB (*n* = 100)	*p *value
Male gender	20 (74.1)	83 (83)	0.405
Age (years)	15.8 (14.6–17.8)	15.9 (14.4–16.8)	0.393
Haller index	3.7 (3.2–4.4)	3.5 (2.9–4.3)	0.256
Funnel depth (cm)	3 (2.5–3.7)	3.1 (2.6–4)	0.721
Symmetrical PE	18 (66.7)	65 (67)	1.0
Duration of VB therapy (months)	17 (8–34)	/	/
Secondary diagnoses	14 (51.9)	36 (36)	0.183

Values are given as absolute numbers (with percentages in brackets) for binary and categorical variables and median (and interquartile range (IQR) in brackets) for continuous variables

*PE *pectus excavatum, *VB *vacuum bell

### Short-term outcomes

Operation time was comparable between groups with 90 min in VB (median, IQR 81.3–113) versus 88 min in non-VB (IQR 69.5–104; *p* = 0.154) patients. Likewise, median length of hospital stay was similar between groups (7 days each, IQR 6–10 each; *p* = 0.385). Time spent in ICU was shorter in patients with preoperative VB therapy (median, 1 day, IQR 1–1) compared to patients undergoing direct MIRPE (1 day, IQR 1–3; *p* = 0.007).

Non-significantly more patients in the VB group needed two bars (18.5% in VB versus 10.2% non-VB; *p* = 0.313). Groups were similar concerning postoperative complications with two (7.4%) in the VB versus eight (8%; *p* = 1.0) in the non-VB and bar dislocations with two (9.1%) in the VB versus eight (8.6%; *p* = 1.00) in the non-VB group. Postoperative complications comprised pneumothorax (three), pleural effusion (three), atelectasis (three), pneumonia (one), restriction in lung function (one), postoperative fever (one) and arrhythmia (one). Median time to bar removal was 35.5 (IQR 28.2–37) months in VB and 36 (IQR 32.2–38.4) months in non-VB patients (*p* = 0.174). Table 2 depicts more details concerning MIRPE.

**Table 2:** Details of minimal-invasive repair of pectus excavatum

Variable		VB (*n* = 27)	Non-VB (*n* = 100)	*p *value
OR time (min)		90 (81.3–113)	88 (69.5–104)	0.154
Length of hospital stay (days)		7 (6–10)	7 (6–10)	0.385
Intensive care unit (ICU)		25 (92.6)	81 (81.8)	0.240
Duration on ICU (days)		1 (1–1)	1 (1–3)	0.007
Chest tube		4 (14.8)	29 (29.3)	0.147
Duration of chest tube (days)		4 (2–6)	3 (1.5–4)	0.439
Postoperative complications		2 (7.4)	8 (8)	1.0
Bar dislocation		2 (9.1)	8 (8.6)	1.0
Median time to bar removal (months)		35.5 (28.2–37)	36 (32.2–38.4)	0.174
Bar removed		23 (100)	98 (99)	1.0
Side of thoracoscopy				1.0
Right		27 (100)	96 (98)	
Left		0	0	
Both		0	2 (2)	
Side of stabilizer				0.218
Right		1 (3.8)	2 (2)	
Left		18 (69.2)	53 (54.1)	
Both		6 (23.1)	41 (41.8)	
None		1 (3.8)	2 (2)	
Sternal elevation during operation		23 (85.2)	67 (68.4)	0.096
Number of bars				0.313
1		22 (81.5)	88 (89.8)	
2		5 (18.5)	10 (10.2)	
Attachment to				0.274
Rib		9 (33.3)	46 (46.9)	
Muscle		18 (66.7)	52 (53.1)	

Values are given as absolute numbers (with percentages in brackets) for binary and categorical variables and median (and interquartile range (IQR) in brackets) for continuous variables

*OR time *operation time, *ICU* intensive care unit

### Long-term follow-up

Long-term follow-up was conducted 7.4 years (median) after primary surgery in VB and after nine years in non-VB patients. The overall rating of the primary operation (MIRPE and hospital stay) was similar between groups (*p* = 0.113) and was rated “very good” by eight patients (50%) in the VB and 42 (67.7%) in the non-VB and “good” by five (31.3%) in the VB and 17 (27.4%) in the non-VB group. Likewise, the perception of pain was similar between groups (*p* = 0.838) and was rated “intolerable” by four patients (23.5%) in the VB and by 10 (16.1%) in the non-VB group. Satisfaction with the cosmetic result was similarly high (*p* = 0.545) in both groups: fifteen (93.8%) patients with the VB versus 47 (75.8%) in the non-VB group reported a “much better” look of their chest area following MIRPE. Table 3 reports on long-term follow-up data.

**Table 3:** Long-term follow-up

Variable	VB (*n* = 17)	Non-VB (*n* = 62)	*p *value
Follow-up after primary surgery (months)	89.1 (60.5–145)	108.2 (71.5–174.1)	0.299
VB therapy offered	16 (94.1)	48 (81.4)	0.279
Rating of primary operation			0.113
Very unsatisfactory	0	1 (1.6)	
Unsatisfactory	0	0	
Acceptable	3 (18.8)	2 (3.2)	
Good	5 (31.3)	17 (27.4)	
Very good	8 (50)	42 (67.7)	
Pain at primary operation			0.838
Intolerable	4 (23.5)	10 (16.1)	
Extreme	11 (64.7)	37 (59.7)	
Moderate	2 (11.8)	10 (16.1)	
Mild	0	4 (6.5)	
None	0	1 (1.6)	
Own perspective of the look of chest			0.545
Much worse	0	0	
Worse	0	1 (1.6)	
No change	0	1 (1.6)	
Better	1 (6.3)	13 (21)	
Much better	15 (93.8)	47 (75.8)	
Feedback from family/friends concerning the look of chest			1.00
Much worse	0	0	
Worse	0	0	
No change	0	2 (3.8)	
Better	1 (6.7)	6 (11.3)	
Much better	14 (93.3)	45 (84.9)	
Posture			0.812
Much worse	0	0	
Worse	0	0	
No change	2 (12.5)	12 (19.4)	
Better	6 (37.5)	25 (40.3)	
Much better	8 (50)	25 (40.3)	
Satisfaction with procedure			0.409
Regret it very much	0	1 (1.6)	
Regret it	1 (5.9)	4 (6.5)	
Neutral	0	0	
Satisfied	0	9 (14.5)	
Very satisfied	16 (94.1)	48 (77.4)	
Time of primary operation			0.583
Too early	0	3 (4.8)	
Correct	10 (62.5)	43 (69.4)	
Too late	6 (37.5)	16 (25.8)	
Intension of doing surgery again	15 (93.8)	54 (90)	1.00
VB therapy after bar removal	0	4 (6.6)	0.574

Values are given as absolute numbers (with percentages in brackets) for binary and categorical variables and median (and interquartile range (IQR) in brackets) for continuous variables

*VB *vacuum bell

### Long-term perception of vacuum bell therapy

Concerning VB therapy, eight (47.1%) patients reported complications, however, limited to skin irritations or hematoma. Only one patient (5.9%) had the impression that VB treatment had delayed surgical treatment, and six (37.5%) patients would have tried preoperative VB therapy again. All patients reported that the main reason for the discontinuation of VB therapy was the lack of improvement. Table 4 illustrates long-term follow-up on VB therapy specifics.

**Table 4:** Long-term follow-up on VB therapy specifics

Variable	VB (*n* = 17)
Complications	8 (47.1)
VB therapy delayed surgical treatment	1 (5.9)
Wish for direct operation	10 (62.5)

Values are given as absolute numbers (with percentages in brackets)

*VB *vacuum bell

## Discussion

Failed vacuum bell therapy had no or minimal effect on short-term outcomes following minimal-invasive repair of pectus excavatum. Likewise, long-term perception of MIRPE and its perioperative period was not affected by preoperative VB therapy. While only 5.9% of patients had the impression that VB treatment had delayed surgical treatment, 37.5% would have tried preoperative VB therapy again.

Our study population included all consecutive patients undergoing a Nuss procedure at our institution over a time course of 16 years. Nevertheless, all the limitations of a retrospective analysis apply. Our study has a relatively small sample size, especially in the VB group. This could inadvertently lead to a type II error regarding postoperative complications. The overall low rates of postoperative complications in our study population, similar to other recent larger scale studies [18–20], require a very large study to confirm our preliminary findings. Decisions made during this study are based on clinical reasoning and dependent on the experience of the surgeon. E.g., the application of a VB therapy is a combined decision involving surgeons, patient and often patients’ caregivers. Furthermore, VB therapy is prone to bad compliance, and compliance to preoperative VB therapy was not assessed in the present study. Follow-up over phone was conducted 9 years (median) after primary operation, which makes it prone to recall bias. Ten (37%; VB group) and 38 (38%; non-VB group) patients were lost to follow-up. Reasons included lack of interest, outdated contact information or death.

The present study assessed a possible effect of failed vacuum bell therapy on MIRPE for the first time. Preoperative VB therapy could potentially have delayed surgical repair. However, our data show comparability of both groups concerning age at operation (median, 15.8 years in the VB group and 15.9 in the non-VB). The recommended age of repair is 12–14 years by its inventor Donald Nuss [10]. Others recommend the repair between 12 and 16 years [7] or 10 and 14 years [21]. The benefit of conducting MIRPE at an early age is the more flexible chest wall and the existence of a bar during the growth spurt in puberty. However, MIRPE can also be performed in adults [6, 22].

On the other hand, one could assume that preoperative VB therapy affects funnel depth and Haller index. Both parameters were almost identical between groups in the present study cohort. While the effect of preoperative VB treatment was not quantified in the present study, all patients in the VB group discontinued VB therapy due to lack of treatment effect. Median duration of VB therapy was 17 months in the present study, exceeding the recommended 12–15-month course of treatment by Haecker and Mayr [12]. This might be explained due to the absent treatment effect and therefore prolonged therapy.

Minimal invasive correction of PE is generally considered a safe and very effective operation and has become standard practice in children with moderate to severe disease for many years [18–20, 23, 24]. However, surgeons must bear in mind, that severe complications such as cardiac tamponade [25], (near)-fatal hemorrhage [26] or postpericardiotomy syndrome [27] can occur. Parents and patients must be counseled accordingly when obtaining informed consent for surgery. Vacuum bell therapy reflects a treatment option for patients with mild/moderate disease and/or patients refusing operative treatment. While evidence for VB therapy is sparse, success rates seem to be higher in younger patients (below 11 years), with minimal or moderate disease (i.e. initial chest wall depth < 1.5 cm) [13, 28]. Reported complications in the literature comprise subcutaneous hematoma, petechial bleeding, dorsalgia, and momentary paresthesia of the arms during application [14]. In our cohort, eight (47.1%) VB patients reported side effects, of whom four suffered from a hematoma, three from skin irritations and one reported an accentuation of a pigment disorder.

## Conclusion

Failed preoperative vacuum bell therapy had no or minimal effect on short-term outcomes and long-term perceptions following minimally invasive repair of pectus excavatum. Vacuum bell therapy can, therefore, be offered safely as a first-line therapy for patients with severe pectus excavatum needing later surgery. Further large-scale studies are, however, warranted to confirm these preliminary results.
