# Correction to: Failed preoperative vacuum bell therapy does not affect outcomes following minimally invasive repair of pectus excavatum

**DOI:** 10.1007/s00383-022-05087-1

**Published:** 2022-03-02

**Authors:** J. L. Muff, L. C. Guglielmetti, S. J. Gros, L. Buchmüller, G. Frongia, F.-M. Haecker, S. G. Holland-Cunz, T. de Trey, Raphael N. Vuille-dit-Bille

**Affiliations:** 1 Department of Pediatric Surgery, University Children’s Hospital of Basel, Spitalstrasse 33, 4056 Basel, Switzerland; 2 Department of Visceral and Thoracic Surgery, Cantonal Hospital of Winterthur, Winterthur, Switzerland; 3 Division of Pediatric Surgery, Department of General, Visceral and Transplantation Surgery, University Hospital Heidelberg, Heidelberg, Germany; 4 Department of Pediatric Surgery, Children’s Hospital of Eastern Switzerland, St. Gallen, Claudiusstrasse 6, 9006 St. Gallen, Switzerland; 5 Faculty of Medicine, University of Basel, 4056 Basel, Switzerland

## Correction to: Pediatric Surgery International (2021) 37:1429–1435 10.1007/s00383-021-04963-6

In the original publication, author name Frank-Martin Haecker and the following affiliation was missing.

^4^Department of Pediatric Surgery, Children’s Hospital of Eastern Switzerland, St. Gallen, Claudiusstrasse 6, CH-9006 St. Gallen, Switzerland

^5^Faculty of Medicine, University of Basel, 4056 Basel, Switzerland.

The original article has been corrected.

